# EFEMP1 suppresses malignant glioma growth and exerts its action within the tumor extracellular compartment

**DOI:** 10.1186/1476-4598-10-123

**Published:** 2011-09-28

**Authors:** Yuanjie Hu, Peter Dion Pioli, Eric Siegel, Qinghua Zhang, Jodi Nelson, Abhishek Chaturbedi, Marlon S Mathews, Daniel I Ro, Selma Alkafeef, Nelson Hsu, Mark Hamamura, Liping Yu, Kenneth R Hess, Bruce J Tromberg, Mark E Linskey, Yi-Hong Zhou

**Affiliations:** 1 Department of Biological Chemistry, University of California Irvine, 5171 California Ave., Suite 150, Irvine, CA (92697), USA; 2 Department of Biostatistics, University of Arkansas for Medical Sciences, 4301 W. Markham St., Little Rock, AR (72205), USA; 3 National Engineering Center for Biochip at Shanghai & Shanghai Biochip Co., Lt, 151 Libing Rd, Shanghai (201203), China; 4 Neurological Surgery, University of California Irvine, 5171 California Ave., Suite 150, Irvine, CA (92697), USA; 5 Tu & Yuen Center for Functional Onco-Imaging, University of California Irvine, 5171 California Ave., Suite 150, Irvine, CA (92697), USA; 6 Ziren Research LLC, 9841 Irvine Center Drive, Irvine, CA (92618), USA; 7 Department of Biostatistics, University of Texas M. D. Anderson Cancer Center, 1515 Holcombe Blvd, Houston, TX (77030), USA; 8 Beckman Laser Institute, University of California Irvine, 5171 California Ave., Suite 150, Irvine, CA (92697), USA

## Abstract

**Purpose:**

There are conflicting reports regarding the function of EFEMP1 in different cancer types. In this study, we sought to evaluate the role of EFEMP1 in malignant glioma biology.

**Experimental Design:**

Real-time qRT-PCR was used to quantify *EFEMP1 *expression in 95 glioblastoma multiforme (GBM). Human high-grade glioma cell lines and primary cultures were engineered to express ectopic EFEMP1, a small hairpin RNA of EFEMP1, or treated with exogenous recombinant EFEMP1 protein. Following treatment, growth was assayed both *in vitro *and *in vivo *(subcutaneous (s.c.) and intracranial (i.c.) xenograft model systems).

**Results:**

Cox regression revealed that EFEMP1 is a favorable prognostic marker for patients with GBM. Over-expression of EFEMP1 eliminated tumor development and suppressed angiogenesis, cell proliferation, and VEGFA expression, while the converse was true with knock-down of endogenous EFEMP1 expression. The EFEMP1 suppression of tumor onset time was nearly restored by ectopic VEGFA expression; however, overall tumor growth rate remained suppressed. This suggested that inhibition of angiogenesis was only partly responsible for EFEMP1's impact on glioma development. In glioma cells that were treated by exogenous EFEMP1 protein or over-expressed endogenous EFEMP1, the EGFR level was reduced and AKT signaling activity attenuated. Mixing of EFEMP1 protein with cells prior to s.c. and i.c. implantations or injection of the protein around the established s.c. xenografts, both significantly suppressed tumorigenicity.

**Conclusions:**

Overall, our data reveals that EEFEMP1 suppresses glioma growth *in vivo*, both by modulating the tumor extracellular microenvironment and by altering critical intracellular oncogenic signaling pathways.

## Background

Fibulins are a seven-member family of secreted glycoproteins, which are characterized by repeated epidermal growth-factor-like domains and a unique *C*-terminal structure [1]. Recent studies exploring the role of fibulins in cancer biology have yielded conflicting results. Different members of the fibulin family have been shown to demonstrate either tumor-suppressive or oncogenic activity [2]. Paradoxically, an individual fibulin can also demonstrate either tumor-suppressive or oncogenic behavior tied to tissue-specific expression. An example of this phenomenon is fibulin 3, officially named EGF-containing fibulin-like extracellular matrix protein 1 (EFEMP1).

In support of a possible tumor-suppression role, EFEMP1 was discovered to have an anti-angiogenic function via suppression of endothelial cell sprouting [3]. There are additional reports showing that: (A) tumorigenicity of fibrosarcoma cells was inhibited by EFEMP1 over-expression, (B) reduced *EFEMP1 *expression and/or *EFEMP1 *promoter methylation occurs in lung, liver, breast, prostate, and nasopharyngeal carcinomas [4-9], and (C) a decrease in EFEMP1 expression in hepatocellular and nasopharyngeal carcinoma is correlated with a worse prognosis [5,9]. In contrast, a potential cancer-promoting function of EFEMP1 was implied in two clinical studies; in one study, the level of EFEMP1 expression was correlated to poor prognosis for cervical cancer [10], while the other study demonstrated *EFEMP1 *over-expression in breast carcinoma [11]. In addition, pancreatic adenocarcinoma cells, EFEMP1 over-expression was shown to promote xenograft formation [12]. The potentially variable tissue-specific effects of EFEMP1 on cancer patient prognosis are reflected in the corresponding tissue-derived cancer *in vitro *assays, revealing the ability of EFEMP1 to either activate [13] or suppress [9] AKT signaling activity in pancreatic or nasopharyngeal carcinoma cell lines, respectively.

In glioma cells, EFEMP1 was shown to enhance *in vitro *substrate-specific cell adhesion and promote cell motility and dispersion [14]. However, to date, there has been no *in vivo *study of EFEMP1 effects on human glioma biology. Results from microarray analyses revealed that EFEMP1 is up-regulated by transcription factor PAX6 - a tumor suppressor in malignant gliomas [15-19]. As a protein functioning in the extracellular milieu, given its potential tumor-suppressive role, there is an interest to develop EFEMP1 into a new therapeutic agent for patients with malignant glioma. We thus carried out an in-depth study of EFEMP1 expression as a prognostic marker in the most malignant grade of glioma, glioblastoma multiforme (GBM). We utilized various human malignant glioma cell lines and primary cultures to examine the mechanisms of EFEMP1 tumor suppression. Most importantly we demonstrated an *in vivo *tumor suppression effect of EFEMP1 in both subcutaneous and intracranial xenograft models.

## Materials and methods

### GBM cDNA samples, patient follow-up, and gene expression quantification

We included 95 glioblastoma multiforme (GBM) cDNA samples and patients' overall survival data from our previously described glioma prognosis project [20]. cDNA samples of human glioma cell cultures and subcutaneous (s.c.) xenografts were made from 2-3 μg total RNA using superscript reverse transcriptase II (Invitrogen). Real-time qRT-PCR were carried out in a StepOne real-time PCR instrument (Applied Biosystems, Foster City) using

AqRT-PCR Standard-1020 (for *EFEMP1*, *VEGFA*) and Standard-1057 (for *KDR*) and primer sets for the marker/target gene and reference gene (*ACTB*), provided by Ziren Research LLC (Irvine, CA).

### Glioma cell lines and primary cultures

High-grade glioma cell lines U251HF, SNB19 and LN229 were gifts from A. Yung's lab at M.D. Anderson Cancer Center [17]. Glioma primary cultures were derived from patient glioma specimens requested and cultured according to approved IRB and IBC protocols. Single-cell suspensions were prepared by digesting tumor pieces with 0.05% Trypsin-EDTA for 30-45 min followed by mechanical dissociation (passing through a glass pipette until smooth) in cold tissue-dissociation buffer (DMEM/F12 containing 0.10 mg/ml DNase and 10% fetal bovine serum). Dissociated cells were cultured in DMEM/F12 medium supplemented with 5% bovine serum in collagen-coated (2 μg/cm^2^) culture plates. All cultures were grown at 37°C in a tissue culture incubator with 5% CO2.

### Plasmid and lentiviral vectors, transfection and infection, and EFEMP1 protein

**Figure 1:**
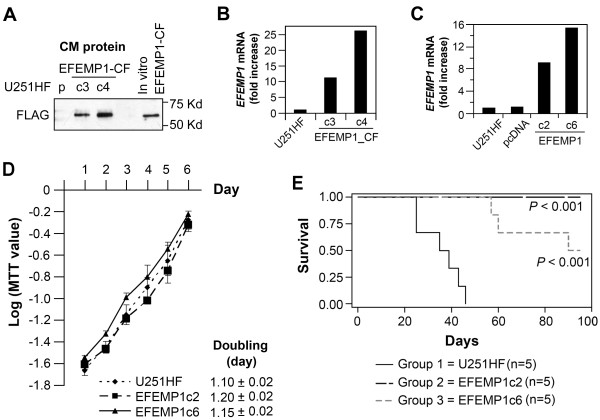
**EFEMP1 over-expression suppressed U251HF cell growth *in vivo *not *in vitro***. **A**, FLAG immunoblot of conditioned-medium (CM) proteins from cell cultures of U251HF (p) and stable transfectants of FLAG-tagged EFEMP1. **B & C**, real-time qRT-PCR quantification of *EFEMP1 *mRNA levels in U251HF transfected with FLAG-tagged or untagged EFEMP1 constructs, normalized to *GAPDH*, and compared with untransfected cell arbitrarily set to unity. **D**, MTT detection of cell *in vitro *growth speed. **E**, Kaplan-Meier survival curves for mice after i.c. implantation with U251HF and its EFEMP1 transfectants.

Full-length EFEMP1 cDNA of protein-coding region by all three transcription variants (NM_001039348, NM_001039349, NM_004105) was PCR-amplified using a 5' primer that contains a *Hin*d III site and a 3' primer with or without an octapeptide FLAG in front of the stop codon. The PCR fragment was then cloned into pcDNA3.1+ and verified by sequencing. The stable EFEMP1-transfected clones of U251HF were established via transfection of plasmid DNA of EFEMP1/pcDNA3.1+ or EFEMP1-CF (*C*-terminal FLAG tag)/pcDNA3.1+. Positive clones were verified by real-time qRT-PCR and immunoblotting with FLAG antibody (Figure 1).

Plasmid constructs of three VEGFA isoforms, VEGF-121, VEGF-165, VEGF-189, and control-vector LacZ were kindly provided by Dr. Shi-Yuan Cheng [21]. Each plasmid construct was transfected into the EFEMP1-transfected clone (EFEMP1c6) of U251HF, and subjected to dual selection with neomycin (400 mg/ml) and hygromycin B (75 mg/ml) for a duration of 2-3 weeks. Lentiviral vectors pGIPZ-shEFEMP1 (expressing a small hairpin RNA of EFEMP1) and pGIPZ-Empty were procured from Open Biosystems (Huntsville, AL). Infectious lentivirus was produced by co-transfection of the lentiviral vector construct with packaging plasmid psPAX2 and envelope plasmid pCMV-VSVG in HEK-293T cells, following the manufacturer's protocol. The infected glioma primary cultures were selected for 1-2 weeks in culture medium containing 1.25 μg/ml puromycin prior to analysis.

Human recombinant EFEMP1 protein was from Abnova (Walnut, CA). Protein was dissolved in vehicle (50 mM Tris-HCl, 10 mM reduced Glutathione, pH = 8.0).

### *In vitro *cell and *in vivo *tumor growth assays

We used an MTT assay to measure glioma cell proliferation *in vitro *previously described, which provides results consistent with viable-cell counting [18]. For formation of intracranial (i.c.) xenografts, cells (1 × 10^5 ^/3 μl DMEM/F12) were injected into the frontal lobe of 4-6 week old female nude mice (stain NCrNu-M, Taconic, Hudson, NY), using a Harvard Apparatus Model 11 Plus Syringe Pump and a mouse stereotactic frame. Mice were observed daily until moribund signs (hunchback and motionless) appeared, and were terminated the following day (which was recorded as the survival date). For formation of subcutaneous (s.c.) xenografts, cells (2.5 or 1 × 10^6 ^cells/50 μl DMEM/F12) were subcutaneously injected into nude mice anterior to their right and left thighs on both sides. Tumor measurements were taken every 3-4 days after implantation, and tumor volume was calculated using the formula V = (L*W^2^)/2 (L, length; W, width).

### Modulated imaging (MI) and magnetic resonance imaging (MRI)

Mice with s.c. xenografts were subjected to modulated imaging as described previously [19], which provides quantitative measures of the *in vivo *concentrations of oxyhemoglobin (OHb), deoxyhemoglobin (RHb) and total hemoglobin (THb). THb is an index of angiogenesis, while oxygen saturation (S_t_O_2_), the ratio of OHb to THb, reflects tumor-cell metabolic activity.

Mice with i.c. xenografts were anesthetized using 50 mg/kg sodium pentobarbital, and transported inside sterile cylindrical mouse-isolation containers that have small exhaust holes that allow the administration of gas anesthesia during the imaging studies. Isoflurane was used for anesthesia during imaging. MR images were acquired using a 7T small animal imaging system. T2-weighted images were acquired using a 2D spin-echo pulse sequence with the following parameters: TR = 3.5 s, TE = 50 ms, matrix size = 256 × 256, FOV = 30 mm, slice thickness = 0.8 mm, NEX = 2. T1-weighted images were acquired using a 2D spin-echo pulse sequence with the following parameters: TR = 350 ms, TE = 10 ms, matrix size = 256 × 256, FOV = 30 mm, slice thickness = 1 mm, NEX = 6. 0.2 mmol/kg of gadodiamide contrast agent were injected *i.v*. into the animal prior to MR imaging. SMIS NMR software was used to calculate the dimensions of the brain tumor in the i.c. xenograft mice. Tumor volume measurement (Volume = Area × Depth × 1/2 in mm^3^) involved calculating the area of the image with largest tumor size by the "free hand" ROI option in the software, which highlights the perimeter of the tumor in that slice. Depth was calculated by multiplying the number of slices where visible tumor was seen by 0.8 (thickness of each slice).

### Immunofluorescence assay

Immunofluorescence of s.c. xenograft cryosections (10 μm) was carried out using PECAM-1 [CD31] (1:300, Millipore Corp, Temecula, CA) and Ki67 (1:1000, Abcam Inc, Cambridge, MA) primary antibodies. Fluroscein anti-rat IgG (H+L) and rhodamine anti-rabbit IgG (H+L) were used as secondary antibodies. The fluorescence signals were detected using IMAGEJ 1.42 (NIHIMAGE) which provide counting (blood vessel number) and total area (size of total blood vessels in a microscope image). The blood vessel density (BVD) was computed based on the vessel number per image of 1.02 mm^2 ^for 4-6 areas from three s.c. xenografts. For the *in vivo *tumor-cell proliferation index (PI), information was presented as the percentage of positive Ki67-staining cells to the total number of DAPI-staining nuclei.

### Western blotting, VEGFA enzyme immunometric assays

Conditioned medium (CM) was harvested from 48-hour cell cultures and spun through Vivaspin 20 columns to increase protein concentration. The TnT T7 Quick Transcription/Translation System (Promega, Madison, WI) was used to synthesize EFEMP1-FLAG protein, which was used as a positive control in immunoblotting for detection of EFEMP1-FLAG using a FLAG M2 monoclonal antibody (Sigma, St. Louis, MO, 1:1000 dilution). Proteins of cell culture and s.c. xenografts were extracted in radioimmunoprecipitation assay (RIPA) buffer containing 1X protease inhibitor cocktail (Roche) with a glass homogenizer (for tumors), and subjected to western blotting. Antibodies used include Fibulin-3 (mab3-5) from Santa Cruz Biotechnology (Santa Cruz, California), Actin from EMD Bioscience (San Diego, CA), EGFR, AKT, and pAKT(Ser 473) from Cell Signaling Technology (Danvers, MA). A human VEGFA enzyme immunometric assay (ELISA) kit (Assay Designs, Ann Arbor, MI) was used for quantification of VEGFA isoform VEGF165 in tumor protein extract according to the manufacturer's instructions.

### Antibody array

Protein microarray analysis were carried out using a phospho-specific antibody microarray (Full Moon Biosystems, Inc) according to the manufacturer's protocol. Briefly, 100 μg of cell lysate in 50 μL of reaction mixture were labeled with 1.43 μL biotin in 10 μg/μL N,N-dimethyformamide. The resulting biotin-labeled proteins were diluted 1:20 in coupling solution before applying to the array for conjugation. The antibody microarray was blocked with blocking solution for 30 min at room temperature, rinsed with Milli-Q-grade water for 3 min, dried with compressed nitrogen, and incubated with the biotin-labeled cell lysates at 4°C overnight. The slide was washed twice with 60 mL of 1× wash solution for 10 min. The conjugated-labeled protein was detected using Cy3-streptavidin. The images and data were acquired on Axon GenePix scanner. Phospho-specific antibody activities were compared by corresponding total protein.

### Statistical analysis

Data was examined prior to analysis for adherence to distributional assumptions, and transformed if necessary via logarithmic transformation to stabilize variance. One-way ANOVAs with standard *post hoc *comparisons were used to identify effects of EFEMP1 on cell MTT value, tumor weight, blood-vessel density, proliferation index, and gene expression between multiple stable transfectants and the untransfected cells. Mixed-models ANOVAs were used to analyze longitudinally measured tumor volumes for effects due to knock-down of EFEMP1 using shRNA and rescue by exogenous EFEMP1 protein. Group average tumor volumes ± standard errors (SEs) were calculated as the grand mean of average volumes across all time points. Doubling times and SEs were calculated from log_2_-scale growth rates and their SEs by inversion and delta method, respectively. Overall survivals in mice implanted i.c. with different EFEMP1 transfectants were estimated via Kaplan-Meier curves and compared for differences via logrank test. Overall survivals in humans with GBM were correlated with EFEMP1 expression in their tumor cDNA samples by means of Cox regression, with the univariate predictor being the log10-scaled ratio of *EFEMP1 *vs. *ACTB *mRNA as measured by absolute real-time qRT-PCR. SAS versions 9.1.3 and 9.2 (The SAS Institute, Cary, NC) were used for all analyses.

## Results

### EFEMP1 expression in GBM is related to a longer overall survival of patients

We investigated the prognostic value of *EFEMP1 *based on its expression in 95 GBM specimens. The mRNA levels of *EFEMP1 *and reference gene *ACTB *were quantified using absolute real-time qRT-PCR [22]. The log10-transformed ratio of *EFEMP1 *to *ACTB *was used as the univariate predictor in a Cox regression with overall survival time as outcome variable. The parameter estimate for *EFEMP1 *expression in GBM was -0.182 [likelihood-ratio P = 0.097], equivalent to a 17% decrease in mortality with each 10-fold increase in the *EFEMP1*/*ACTB *ratio, which suggests that the overall survival time increases as EFEMP1 expression levels increase. This finding suggested that EFEMP1 may be important to study for its tumor suppressor function in GBM.

### EFEMP1 suppresses tumorigenicity of human high grade glioma cell line U251HF

We started an investigation of EFEMP1 using the human malignant glioma cell line U251HF, which is highly tumorigenic and forms GBM-like infiltrating necrotic tumors in i.c. xenograft model systems [17]. Since EFEMP1 is a secreted protein within the extracellular matrix compartment, we initially determined the ability of transfected cells to secret a FLAG-tagged EFEMP1 in culture medium. As shown in Figure 1A, western blotting of proteins secreted by cells showed FLAG-tagged EFEMP1. Secreted protein levels correlated with *EFEMP1 *mRNA expression quantified by real-time qRT-PCR (Figure 1B).

We focused the functional assays on the U251HF cell line transfected with un-tagged EFEMP1 (EFEMP1c2:9-fold increase of *EFEMP1 *mRNA, EFEMP1c6: 15-fold increase of *EFEMP1 *mRNA, Figure 1C). As shown Figure 1D, there was no obvious effect of EFEMP1 expression on *in vitro *cell growth, nor were there any morphological changes (data not shown). However, EFEMP1 over-expression dramatically suppressed tumorigenicity (Figure 1E). The survival of mice after i.c. implantation of the EFEMP1-transfected cells was significantly prolonged (EFEMP1c6) and in some instances, animals were completely free of tumor (EFEMP1c2). As expected, animals implanted with the un-transfected parental cells (U251HF) all died of their tumor between 24-46 days post-implantation. Two of the five mice implanted with EFEMP1c6 cells died between 57-60 days, one died at 90 days, while the remaining two survived beyond 95 days after implantation. A repeat of the i.c. implantation experiment confirmed EFEMP1-mediated suppression of U251HF tumorigenicity in the *in vivo *i.c. xenograft model system.

### EFEMP1 suppresses VEGFA-induced angiogenesis

The strikingly different effect of EFEMP1 on glioma-cell growth potential *in vitro *and *in vivo *suggested involvement of tissue or environmental factors, and we strongly suspected angiogenesis to play a key role. To evaluate this possibility, we implanted the same groups of cells tested for tumorigenicity in i.c. locations into subcutaneous (s.c.) locations of nude mice. This allowed for the dual monitoring of tumor growth speed and measurements reflecting the blood uptake and consumption by tumors in live animals using modulated imaging [19].

**Figure 2:**
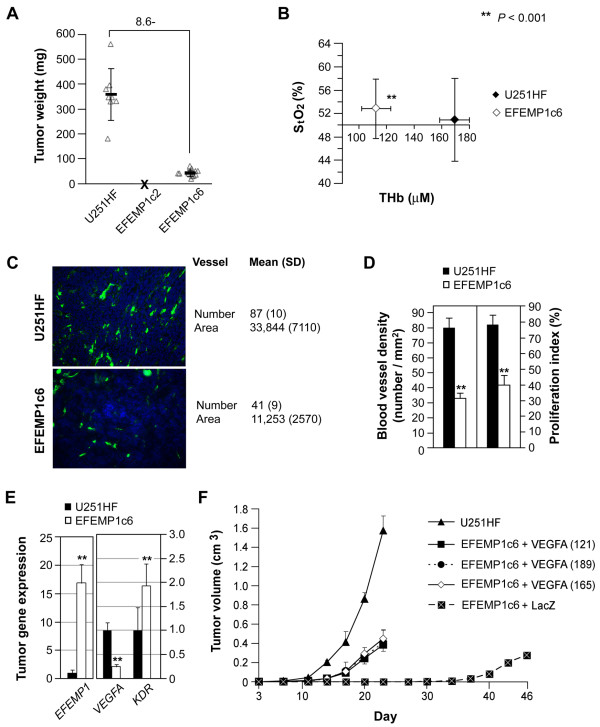
**EFEMP1 suppression of tumorigenicity, *in vivo *cell proliferation, and VEGFA-induced angiogenesis**. **A**, weights of s.c. xenografts dissected 24 days after implantation with 5 × 10^6 ^cells of U251HF and its EFEMP1 transfectants. **B**, total (THb) hemoglobin concentration and tissue oxygen saturation (S_t_O_2_) of s.c. xenografts in live mice by modulated imaging before tumor dissections. **C-D**, immunofluorescence of tumor frozen-sections, with CD31 antibody detecting blood vessel density and Ki67 antibody detecting proliferation index after DAPI-counterstaining of the nuclei. **E**, real-time qRT-PCR quantification of gene expressions in s.c. tumors above, with mean and SD for 6-9 tumors, normalized to *ACTB*. **F**, tumor growth curve based on tumor volume measurement after s.c. implantation.

EFEMP1 over-expression in U251HF cells led to tumor growth suppression of s.c. tumors just as it had for i.c. tumors. EFEMP1c2 failed to form any tumors in 3 months, while tumor size at day 24 for EFEMP1c6 was 12% of the average tumors derived from the parental cells (Figure 2A). Results from modulated imaging revealed that the tumors with EFEMP1 over-expression had significantly lower levels of both OHb and RHb compared to parental cell-derived tumors, resulting in significantly lower levels of THb. No significant difference in S_t_O_2 _was observed (Figure 2B). This data suggested that increasing EFEMP1 expression in U251HF inhibited angiogenesis but not oxygen-dependent metabolic activity.

Immunofluorescence detection of mouse endothelial cell marker CD31 antigen in s.c. tumor sections revealed a significant loss in number (average 41%) as well as a reduction in size (average 33%) of blood vessels in s.c. xenograft of EFEMP1c6 vs. U251HF (Figure 2C). The cell proliferation index in EFEMP1c6 tumors was approximately 50% of that in U251HF tumors (Figure 2D), suggesting that restricting angiogenesis retards tumor proliferation and subsequent tumor growth.

Since VEGFA is a dominant pro-angiogenic factor driving GBM angiogenesis, we quantified *VEGFA *mRNA in s.c. tumors and found a significantly lower expression of *VEGFA *in EFEMPc6-derived tumors compared to those from U251HF. In contrast, the expression of VEGFA receptor gene (*KDR*) was significantly higher in EFEMP1c6-derived tumor compared that of U251HF (Figure 2E). The increase in receptor expression may suggest a compensatory mechanism to offset the loss of ligand. The effect of EFEMP1 on the expression level of *KDR *and its physiological relevance requires further exploration.

To determine the degree of VEGFA involvement in EFEMP1-mediated suppression of glioma-cell growth *in vivo*, VEGFA cDNA constructs encoding three VEGFA isoforms (121, 165 or 189) were transfected in EFEMP1c6. As shown in Figure 2F, the tumor onset time (time when the tumor becomes measurable) for all three VEGFA-transfectants was 17 days, close to that of the original U251HF at day 12, whereas the tumor onset time of the lacZ-transfectant is 40 days after implantation. Although over-expression of ectopic VEGFA was able to partially rescue the tumor growth from the suppression by EFEMP1, restoration of tumor growth rates were far from complete. At day 23 after implantation, the average tumor weight from VEGFA-transfected EFEMP1c6 was 24-27% of U251HF. Thus suppression of VEGFA is likely only one of several probable mechanisms underlying EFEMP1 suppression of glioma growth and EFEMP1 exhibits some VEGF-independent anti-tumor effects.

**Figure 3:**
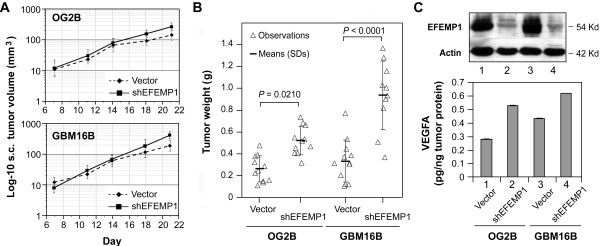
**EFEMP1 knock-down promoted tumorigenicity and VEGFA production by two glioma primary cultures representing tumor mass cell populations**. **A**, growth curves of s.c. xenograft from implantation of 1 × 10^6 ^cells of adherent primary cultures of GBM (GBM16B) and anaplastic oligodendroglioma (OG2B), infected with lentivirus of empty vector or shRNA of EFEMP1 (shEFEMP1). **B**, weights of above tumors dissected 28 days after implantation. **C**, immunoblotting of protein extracts from above s.c. xenografts with EFEMP1 and Actin antibodies (*upper panel*), and quantification of VEGFA protein level in tumor by an VEGF-165 immunometric assay in duplicates (*bottom panel*).

We then further investigated the effect of reducing EFEMP1 levels (via lentiviral expression of shRNA of EFEMP1 - shEFEMP1) on glioma cell tumorigenicity. The results showed that EFEMP1 knock-down in U251HF cells did not cause significant effects on s.c. tumor growth (data not shown). This result is most likely due to a low basal level of EFEMP1. We then investigated two primary culture lines derived from a WHO grade IV GBM (GBM16B) and a WHO grade III anaplastic oligodendroglioma (OG2B), and showed that EFEMP1 knock-down promoted tumor growth starting at day 14 after s.c. implantation. This led to a significant increase of tumor weight at day 28 (Figure 3A and 3B). Western blot and ELISA analyses of tumor lysates confirmed reduction of EFEMP1 protein levels due to shEFEMP1 expression. Of interest, reduction of EFEMP1 corresponded with an increase of VEGFA (Figure 3C). Noticeably, there were also positive correlations between tumor weight and VEGFA levels for the two primary tumor lines.

### EFEMP1 also targets EGFR and AKT signaling

**Figure 4:**
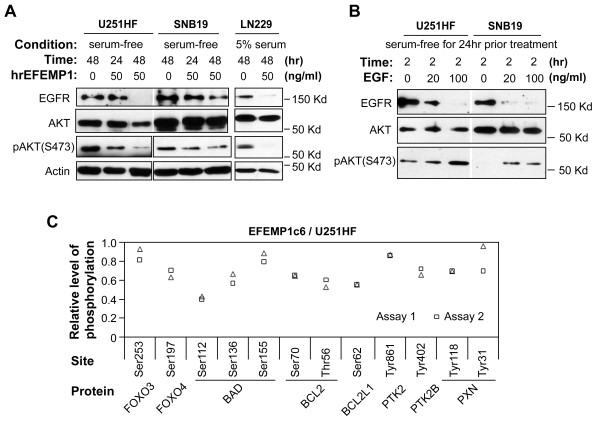
**EFEMP1 suppressed EGFR-AKT signaling**. Whole cell lysate of three human glioma cell lines were immunoblotted with EGFR, pAKT and total AKT antibodies after treatments with EFEMP1 protein or EGF **(B) **for various time periods and in different conditions. **(C)**, protein array analysis of cell lysates of EFEMP1-transfected and un-transfected U251HF following a 48-hour serum starvation, showing relative levels of changes on phosphorylated proteins normalized to the level total proteins.

EFEMP1 contains repeated EGF modules and was shown to activate AKT signaling via binding to EGFR in pancreatic carcinoma cells [13]. However, we observed reductions of total EGFR and phosphorylated AKT (S473) levels after a 48-hour incubation period in three different glioma cell lines using the same purified EFEMP1 protein. As shown in Figure 4A, in the two PTEN null/mutant human glioma cell lines (U251HF and SNB19) under serum-free conditions, the suppressive effect of EFEMP1 on AKT phosphorylation was seen 24 hours after the treatment, while total EGFR reduction occurred a day later. When culturing cells in medium containing 5% bovine serum during EFEMP1 treatment, there was no effect of EFEMP1 on EGFR and AKT phosphorylation (data not shown). For the PTEN wild-type cell line (LN229), pAKT levels were undetectable when cultured in serum-free medium (data not shown), while EGFR and AKT phosphorylation was suppressed by EFEMP1 with serum present. In comparison, 2 hours of exposure to EGF enhanced AKT phosphorylation while reducing EGFR levels (Figure 4B), reflecting the reported negative-feedback regulation of EGFR [23].

We then screened an antibody array using whole-cell lysate of U251HF and its EFEMP1 stable transfectant EFEMP1c6 after a 48-hour serum starvation. EFEMP1 over-expression decreased the phosphorylation levels of multiple AKT substrates (Figure 4C), which is consistent with an AKT-suppressive role for EFEMP1. Substrates affected include FOXO transcription factors (FOXO3 and FOXO4) [24-26] and BCL-2 protein family (BAD, BCL2, and BCL2L1) [27-29] in which the phosphorylated state may ensure glioma cell survival under stressful environments. EFEMP1 also reduced the phosphorylation of PTK2 (known as FAK), PTK2B (known as Pyk2) and PTK2 downstream target PXN (known as Paxillin), which together form the FAK-signaling pathway. FAK has been shown to be upstream of AKT-signaling in promoting malignant behaviors of high grade gliomas [30-32].

### Human recombinant EFEMP1 protein suppresses glioma cell growth *in vivo*

**Figure 5:**
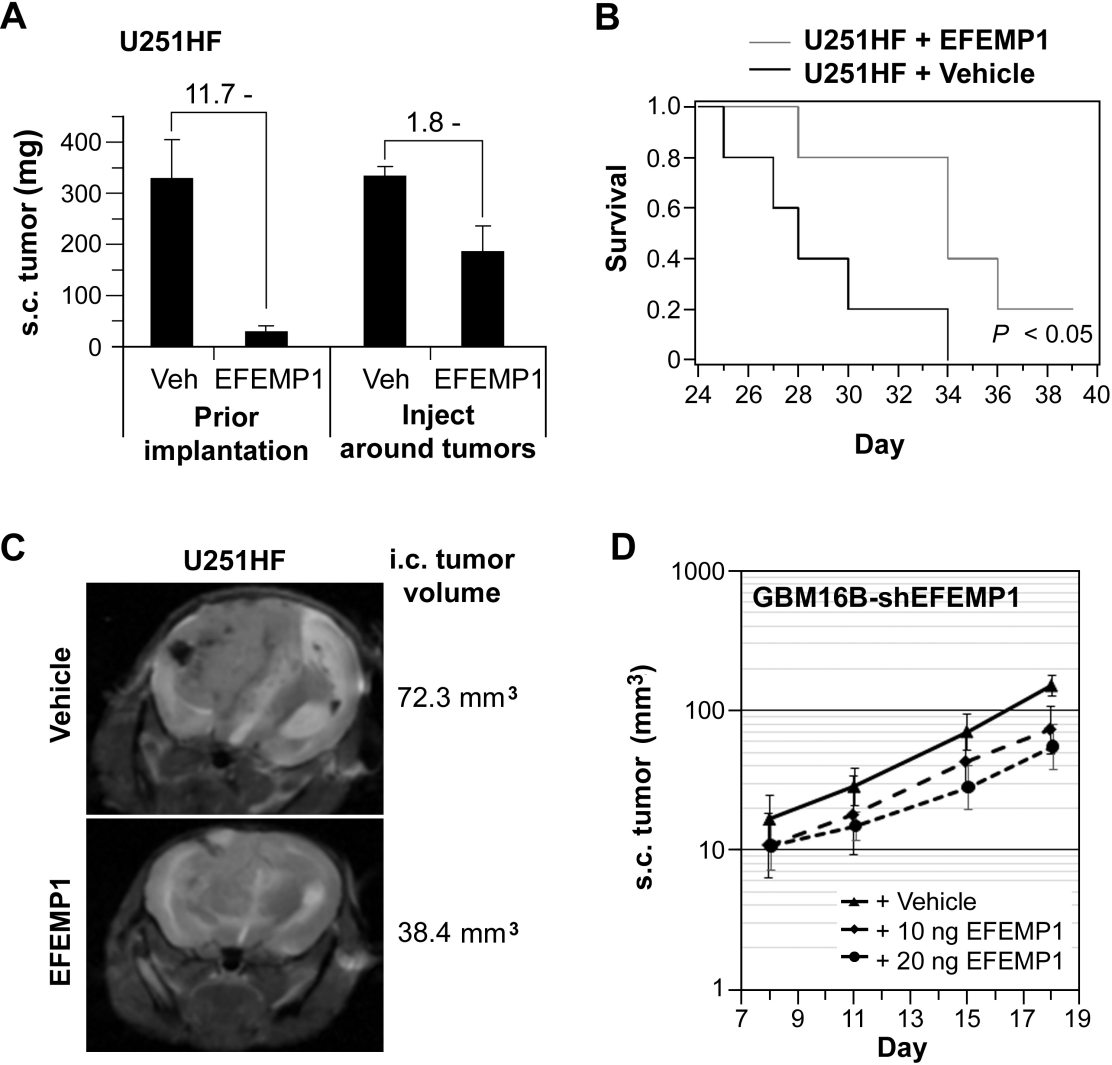
**Human recombinant EFEMP1 protein is able to suppress glioma xenograft growth**. **A**, weight of s.c. xenografts dissected 28 days after implantation of U251HF cells (1 × 10^6^) mixed with EFEMP1 protein (20 ng) or vehicle prior implantation, or peri-tumoral injection with EFEMP1 (10 ng × 2 sides) or vehicle 1 week after implantation and a repeat of EFEMP1 treatment in the following week. Mean (SD) are based on 4 tumors with treatments per group. **B**, Kaplan-Meier survival curves for mice after i.c. implantation with U251HF cells (1 × 10^5^) mixed with 4 ng EFEMP1 protein or same volume of vehicle. **C**, T2-weighted images of mice from vehicle and EFEMP1-treatment groups 32 days after implantation. **D**, s.c. xenograft growth curve after implantation with the same amount of GBM16B expressing shEFEMP1, mixed with the same volume of EFEMP1 or vehicle prior implantation. Symbols (error bars) on the curves represent averages (SDs) of tumor volumes based on longitudinal data from 8 or 9 tumors per group that was log-transformed to stabilize variances for the repeated-measures ANOVA. *P *< 0.001 for the test of any separation between curves among all three groups, and *P *= 0.046 for the test of any difference in growth rates among all three groups.

Based on the suppressive function of human recombinant EFEMP1 on EGFR and AKT signaling activities, we undertook a study of this protein to see whether it would be capable of suppressing GBM cell growth *in vivo*. There was a significant suppression of tumor development from EFEMP1 via homogenous mixing with cells prior to implantation in both s.c. and i.c. xenograft systems. This effect was also seen when EFEMP1 protein was administrated circumferentially around established s.c. xenografts (Figure 5A-B). These findings after both types of EFEMP1 administration *in vivo *are consistent with our *in vitro *data demonstrating that that EFEMP1 suppresses intracellular oncogenic signaling, and is also consistent with a potential suppressive effect on *in vivo *angiogenesis by directly targeting endothelial cells [3].

Magnetic resonance imaging (MRI) was utilized for temporal comparison of i.c. tumor development without losing survival data. As shown in Figure 5C, the i.c. tumor size in mice implanted with U251HF cells plus EFEMP1 was smaller compared to cells plus vehicle. MRI also revealed differences in i.c. tumor morphologies between EFEMP1 and vehicle treatment. Consistently shown in three individual mice per group, the vehicle group exhibited solid tumors with well-defined boundaries, a mass effect with right to left midline shift, and edema around the tumor. In contrast, the EFEMP1 treatment group exhibited a diffusely infiltrative mass, as is evident by the presence of edema in the right brain without a discernible solid tumor.

In respect to the finding shown in Figure 3 that EFEMP1 knock-down enabled a higher tumorigenicity, we applied EFEMP1 protein to cells expressing shEFEMP1 prior to s.c. injection. As shown in Figure 5D, EFEMP1 protein treatment was able to antagonize the tumor promoting effect from EFEMP1 knock-down in GBM16B. Group averages ± SEs were 47.5 ± 3.6 mm^3 ^in the vehicle treatment group, compared to only 27.8 ± 2.2 mm^3 ^in the +10 ng EFEMP1 treatment group and 22.3 ± 1.7 mm^3 ^in the +20 ng EFEMP1 treatment group (*P *< 0.0001). Tumor doubling times were increased in a somewhat dose-dependent manner, being 3.14 ± 0.23 days with vehicle, 3.55 ± 0.31 days with +10 ng EFEMP1, and 4.24 ± 0.42 days in the +20 ng EFEMP1 (*P *= 0.046).

## Discussion

The overall results of this study revealed a role for EFEMP1 in the suppression of glioma growth, via independent blockade of EGFR and AKT signaling pathways and the repression of VEGFA-induced angiogenesis. Activation of these molecular pathways is a well-known molecular-pathological feature of GBM. The finding of a favorable clinical prognosis effect from *EFEMP1 *expression in GBM patients is consistent with these laboratory findings. EFEMP1 anti-angiogenic effects appear to take place through both VEGFA-dependent and VEGFA-independent mechanisms. EFEMP1-mediated suppression of glioma-cell expression of *VEGFA *would result in the suppression of VEGFA stimulation of angiogenesis, which is in addition to EFEMP1's direct inhibition of endothelial cells sprouting [3].

In addition to the overall improvement of mouse survival resulting from EFEMP1 over-expression, MRI data of i.c. glioma morphologies revealed EFEMP1's function in potentiating glioma-cell infiltration, as shown by *in vitro *studies [14]. This is the first demonstration of EFEMP1's role in the regulation of glioma cell invasion in an i.c. xenograft system. In our GBM cell line/primary culture *in vivo *systems, the tumor-suppressive effect of EFEMP1 overwhelmed its pro-invasive activity. Further investigation is needed to understand the molecular context of EFEMP1 in control of different malignant behaviors of cancer cells.

This study showed that EFEMP1 repressed AKT activity and phosphorylation of multiple AKT substrates in human malignant glioma cells. These results differed from observations in pancreatic adenocarcinoma cells, where EFEMP1 enhanced AKT phosphorylation [13], but were in accordance with findings in nasopharyngeal carcinomas cells, where EFEMP1 suppressed AKT phosphorylation. Such discrepancies may be related to different membrane receptors and downstream signaling pathways given the plethora of molecular and cellular abnormalities in different cancer cell types.

EGFR amplification occurs in 40 to 70% of primary GBMs, but is not observed in lower-grade astrocytomas [33]. Activation of EGFR and PI3K/AKT signaling pathways are clearly the causes of malignant behavior of GBM and emerges as common pathways regulating cellular proliferation, survival, migration and invasion [34]. Our data revealed the negative regulation of EGFR by EFEMP1, via down-regulation of total EGFR levels. It is well-documented that receptor activation by EGF leads to the internalization and degradation of EGFR [35]. The EFEMP1-initiated reduction of total EGFR, although not as quick as EGF-initiated negative-feedback regulation of EGFR, could involve a similar protein-degradation mechanism, since EGFR mRNA levels were not reduced upon EFEMP1 treatment.

We have shown that EFEMP1-mediated down-regulation of AKT signaling occurs in PTEN null/mutant glioma cells under serum-starvation conditions but not in serum-containing medium. This is consistent with the lack of an EFEMP1-mediated effect on cell proliferation in serum-containing culture medium, in contrast to the dramatic suppression of tumor formation in both s.c. and i.c. xenograft systems. Our data revealed that EFEMP1 suppresses tumor growth via multiple mechanisms. In addition to the aforementioned effect on attenuating EGFR/AKT signaling activities, we demonstrate EFEMP1 involvement in regulation of the tumor microenvironment through down-regulation of glioma-cell VEGFA production. The mechanism of EFEMP1 in the down-regulation of *VEGFA *expression is not clarified by this study, though it could be an indirect effect from inhibition of AKT signaling [36].

Current anti-angiogenic agents such as bevacizumab, a humanized VEGFA antibody, have met with limited success in glioma patients due to its promoting cell invasion [37, 38]. Either EGFR-targeting monoclonal antibody or inhibition of tyrosine kinases activity in glioma patients produced minimal tumor response and no improvement in overall survival [39, 40]. The identification for EFEMP1 of a VEGFA-independent mechanism in addition to its VEGFA-dependent mechanism in suppressing angiogenesis and EFGR- and AKT-signaling activities suggests that it may be possible to develop additional anti-angiogenic agents that are complementary to bevacizamab, or potentially still effective in the face of bevacizumab resistance. While more studies are needed to further define the role of EFEMP1 (i.e. anti-angiogenic vs. pro-invasive), results from this study provide a mechanistic rationale on developing EFEMP1, or EFEMP1-derived signaling peptide, as a new potent therapy for patients with high-grade gliomas.

In reviewing published studies on EFEMP1 in cancer and incorporating results from this study, it should be noted that EFEMP1's function, whether promoting or inhibiting cancer growth, may be dependent on a molecular context that differs by cancer cell type and malignancy stage. The identification of proteins that are cooperative with EFEMP1 in the regulation of intracellular signaling pathways and the tumor microenvironment would distinguish the differential effect of EFEMP1 in cancer. The tumor-suppressive effect of EFEMP1 to glioma cells may also be applicable to other cancer types that have hyperactivation of EGFR and AKT signaling pathways. Some of these are lung, liver, breast, and prostate cancers, where *EFEMP1 *promoter methylation and/or expression were down-regulated [4-8].

## Conclusion

Our data revealed that EFEMP1 is a favorable prognostic factor for GBM, has a tumor-suppressive effect in malignant glioma cells, and acts in the extracellular compartment via independent blocking of EGFR and AKT signaling pathways while also repressing VEGFA-induced angiogenesis. Results of these findings justify therapeutic development of EFEMP1-derived agents for GBM. In addition, the discovery of the function of EFEMP1 as a tumor suppressor that modulates EGFR suggests a need to further investigate EFEMP1 as a potential predictive marker for anti-EGFR therapies of cancer, including glioblastoma, lung, breast, prostate, and liver cancers.

## List of abbreviations

BVD: blood vessel density; ELISA: immunometric assay; MI: Modulated imaging; MRI: magnetic resonance imaging; GBM: Glioblastoma multiforme; i.c.: intracranial; s.c.: subcutaneous; Ohb: oxyhemoglobin; RHb: deoxyhemoglobin; THb: total hemoglobin; S_t_O_2_: oxygen saturation; PI: proliferation index.

## Competing interests

The authors declare that they have no competing interests.

## Authors' contributions

YH, PDP, QZ, JN, AC, MSM, DR, SA, NH, MH, and LY have participated in processing the experiments of with data presented in this manuscript and/or verified the conclusions of this study. KRH performed the Cox analysis and data interpretation, ES performed all statistical analyses, BT contributed to the MI data interpretation, and ML participated in data discussion. YHZ conceived this study, made the constructs, participated in its design and coordination. The manuscript was written by YHZ, edited by ES, ML, and PDP. All authors read and approved the final manuscript.

## Author's information

Corresponding author YHZ, assistant professor in UC Irvine since 2006, has started her research focus since her discovery of PAX6 suppressing GBM cell growth *in vivo *and as a favourable prognosis factor in high-grade gliomas and, with mechanisms involved in suppression of glioma invasion, survival under oxidative stress, and angiogenesis published in Clinical Cancer Research in 2003, Cancer Research in 2006, and Journal of Neuro-Oncology in 2005 and 2010. This study is a continuation of her study of PAX6 in glioma, focusing on identification of a therapeutic target/agent for GBM treatment. From the initial discovery of EFEMP1 as a potential target of PAX6 in 2004 while YHZ was research assistant professor in UAMS, her lab focused on experiments designed to explore the role of EFEMP1 as an effector of PAX6 suppression of *in vivo *growth by suppression of angiogenesis, and the clinical prognosis effect of EFEMP1 for the translational meaning of results from this study. Overall 16 researchers participated in this study from a period of 6 years.

First author YH, PhD candidate, UC Irvine, has participated in PAX6 and EFEMP1 studies for 5 years, co-authored in CR and JNO papers from Zhou lab. KRH, professor, MD Anderson Cancer Center, has been collaborating with YHZ since 2003 on glioma prognosis study based on real-time PCR quantified gene expression data.
